# Three Cases of Ataxia

**Published:** 1913-09

**Authors:** J. M. Fortescue-Brickdale

**Affiliations:** Assistant Physician to the Bristol Royal Infirmary, and Clinical Lecturer in the University of Bristol.


### THREE CASES OF ATAXIA.

BY

J. M. Fortescue-Brickdale, M.A., M.D. Oxon.,

*Assistant Physician to the Bristol Royal Infirmary, and Clinical Lecturer in the University of Bristol.*

---

**Case 1.**—*Family History*—Father was a drunkard. Patient has two healthy sisters and one brother, whose history is given below.

*Personal History*—Enteric fever when in India. No history of syphilis. Drank much beer as a young man. Was in the army six years; then worked as a miner until seven years ago, when his legs began to get weak and his speech became affected. He had to give up his work. Arms were affected later. Vague pains in limbs and trunk (the latter said to resemble pleuritic pain) have occurred for last six months and his memory has failed.

*Condition on Admission* (January 2nd, 1909).—Patient's age is now 48. He is well nourished. No obvious wasting of muscles, which move fairly well against resistance. Gait is staggering and of the "cerebellar" type, but Romberg's sign is absent. There is slight spasticity in thigh muscles. Marked inco-ordination in arms. Speech slow, often louder than necessary, with elision of syllables and halting. No sensory disturbances. Knee-jerks brisk. Rectus clonus both sides. Ankle clonus absent. Plantar reflex—right foot flexor; left foot not obtained. Eyes—pupils equal, react to light and accommodation; no nystagmus, but patient cannot fix his eyes on any object for more than a few seconds; no optic atrophy or neuritis. Sphincters unaffected. Electrical reactions—Faradism, normal; galvanism, no R.D.

Patient has improved under treatment, namely rest in bed and liquor arsenicalis. His speech is not so slow and blurred, and he is steadier on his feet. He remained under observation for some months, but was subsequently lost sight of, and his present condition is unknown.

**Case 2.**—The patient's brother, age 41, gives the following history. Has always been healthy; no history of alcohol or syphilis; has never been out of England. Works as a miner. His wife has had four children, now alive, of whom one is "delicate," three have died in infancy of wasting. About ten years ago some inco-ordination was noticed, and five or six years ago his strength failed, and he had to take lighter work. He is now engaged in looking after the wooden beams and rafters in the pit, which is the lightest job he can get underground. His speech has been affected for five or six years. Within the last six months he has had two attacks of pain and swelling in the knee-joint. No history of headache or vomiting.

*Present Condition* (January 24th, 1909).—The inco-ordination of the arms and head is well marked. The movements are distinctly choreiform in character, and there is much grimacing and twitching of the facial muscles when he speaks. Speech is thick and blurred with some elision of syllables and occasional halting; in fact, it closely resembles his brother's. Muscular power is very fair. There is no disturbance of sensation or no urinary or rectal trouble. The knee-jerks are brisk on both sides; no tendon jerks can be obtained in the arms; no rectus clonus; slight ankle clonus (two or three contractions) in right ankle, which is also a little stiff in movement. The feet are cold, but slight flexor plantar reflex can be obtained on right side. The pupils react to light and accommodation. Romberg's sign is absent, and there is but little ataxy in the gait. On the left arm there is a boil.

**Case 3.**—*Family History*—Father has had a stroke, and is paralysed on one side. Mother has had rheumatic fever several times. One sister has had "neuritis" in one arm. Four brothers and four sisters all alive and now well, but all suffer from rheumatic pains at times. There is no history of epilepsy, fits or other constitutional disease in the family.

*Personal History*—At the age of 19 patient had an attack of influenza, but otherwise she was strong and healthy until the onset of the present disease. Menstruation has always been regular and normal in character.

*Present Illness*—At the age of 22 she had an attack of "twitching" of legs and chattering of the teeth, which began suddenly and was accompanied by a feeling of illness and weakness. The attack lasted about twenty minutes, and was followed by several others during the course of the next month. Two years later she had a similar series of attacks of tremor, and with variations in frequency they have continued ever since. The patient has apparently been unable to get about or do any work during the whole time owing to inability to control her movements. The attacks of tremor are worse about the time of her periods, and are induced by over-fatigue, sudden noises, and shocks or excitement of any kind. She sleeps badly.

The patient came to the out-patient department at the Bristol Royal Infirmary in the spring of 1912, and was admitted under my care. I have had her under observation ever since; and though she states that she is better than she was a year ago, I find little evidence of improvement or alteration in her condition.

*Present Condition*—The patient is a well-nourished young woman of 28. The abdominal and thoracic organs are normal; blood pressure, 130; urine, normal. Her muscles are not wasted, and there is no anæsthesia. She has well-marked ataxia of the lower extremities, and less marked ataxia of the arms. She writes with difficulty, and at times cannot dress herself. The gait is ataxic, and there is no marked difference in her power of maintaining equilibrium when her eyes are closed or open. There is no loss of muscular power, nystagmus, or ocular changes. The knee-jerks are exaggerated; the wrist and elbow jerks present; the superficial reflexes are normal. Plantar reflex is flexor. The pupils react to light and accommodation. Under the influence of excitement or exertion she has marked clonic contractions of the arms and legs of a choreiform character. Less violent attacks of tremor also occur. There is no tremor during sleep, and the attacks always diminish when she rests in bed. There is no difficulty in swallowing or disturbance of speech. For the last twelve months the patient has had some difficulty in controlling the action of the bowels and bladder, not quite amounting to incontinence. She has never had retention of urine.

The first two cases may be grouped under the familial ataxias, and most closely conform to those described by Sanger Brown in 1892. This class is distinguished from the ordinary type of Friedreich by the later onset of the symptoms, the exaggeration of the reflexes, the absence of nystagmus, scoliosis and club-foot, and by the frequent presence of optic atrophy. The cases here described differ from Sanger Brown's in the absence of optic atrophy and of drooping of the eyelids and the late age of onset. The second case showed arthropathies and boils, which are not typically present.

The third case, apart from the fact that no familial incidence could be proved, differed from the types of Friedreich and Sanger Brown in the absence of speech defects and difficulty of swallowing. There is also no change in the *fundi oculorum*, and there is some involvement of the bladder and rectum. Nor do the symptoms in the third case correspond at all to any of the forms of adult chorea; the patient's intelligence, for instance, is normal. The group of myoclonias may also be excluded, as the defect is rather an ataxia than a clonus. Provisionally, a diagnosis of disseminated sclerosis seems the most possible, but the further development of the case will require watching in order to arrive at any degree of certainty.

